# Study of the independent gametophytes found on Jeju Island in South Korea and the first record of the obligate independent gametophyte of *Antrophyum obovatum* Baker

**DOI:** 10.1002/ece3.6510

**Published:** 2020-07-07

**Authors:** Sang Hee Park, Jung Sung Kim, Hyoung Tae Kim

**Affiliations:** ^1^ Department of Forest Science Chungbuk National University Chungbuk South Korea; ^2^ Institute of Agricultural Science and Technology Chungbuk National University Chungbuk South Korea

**Keywords:** *Antrophyum obovatum*, independent gametophyte, Jeju island, land bridge

## Abstract

Fern gametophytes have often been neglected in research; however, studies on gametophytes are crucial for a better understanding of the evolution of ferns. During their life cycle, some gametophytes produce large and long‐lived populations without producing sporophytes and reproduce independently through asexual means, such as through the formation of gemmae.

In this study, we investigated independent gametophytes on the Jeju Island of Korea, which was located on the land bridge between East China and Japan during the glacial periods. Fourteen gametophyte populations were collected from seven sites, of which 13 populations were clearly identified as belonging to four fern species known to occur in Jeju Island with BLAST searches using *rbcL* and *trnL‐F* sequences.

Surprisingly, the last remaining population constituted undescribed taxa in Korea. We presented the first report of the independent gametophytes of *Antrophyum obovatum* Baker which has not been previously recorded in Korea. It has been supposed that many ferns sought suitable habitat throughout the land bridge between China and Japan. However, Jeju Island might be unsuitable for vittarioid ferns that prefer a tropical or subtropical environment. Consequently, only two species of vittariod ferns (*A. obovatum* and *Haplopteris flexuosa* (Fée) E.H. Crane) in the form of a gametophyte and sporophyte, respectively, exist on Jeju Island. Therefore, this gametophyte population must be protected and managed from a conservation perspective.

In the case of the independent gametophyte of *Hymenophyllum wrightii* Bosch, haplotype analysis was conducted based on the *rbcL* sequences and the result supported that the North American populations were migrated from Japan through land bridge during the glacial periods and Jeju populations were recently established by long‐distance dispersal of the Japanese populations.

## Graphical abstract

This work is about independent gametophytes in Korea. However, we found the independent gametophyte, *Antrophyum obovatum*, which is firstly reported in this study.

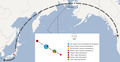

## 1 INTRODUCTION

Land plants experience two‐generation phases during their life cycle, namely the gametophyte and the sporophyte (Kenrick & Crane, 1997). In contrast to Bryophytes whose life cycle is predominantly of the gametophyte generation, plants with vascular tissue systems possess a sporophyte dominant life cycle (Kenrick & Crane, 1997; Qiu, Taylor, & McManus, 2012). Ferns are an early diverging lineage possessing euphylls and a unique reproductive system among vascular plants. Generally, the asexual spores of ferns disperse from the sporophyte leaf and germinate into a gametophyte, which exists independently of the sporophyte. Conversely, the seed plants produce two heterospores, pollen and megagametophytes, which are completely dependent on the sporophyte (Mehltreter, Walker, & Sharpe, 2010).

The gametophyte of ferns has long been neglected in fern research because they are difficult to be identified at species level taxonomy based on the morphological characters (Nayar & Kaur, 1971). Indeed, mature sporophytes of ferns can be generally distinguished according to their morphological characteristics; however, identifying the morphological differences among gametophytes of congeneric species has not been feasible (Ebihara et al., 2013; Imaichi, 2013; Nayar & Kaur, 1971) owing to their being relatively small and simple entities and the lack of specialized organs (Nayar & Kaur, 1971). To classify and recognize the gametophytes, Nayar and Kaur (1971) suggested five types of prothalli for homosporous ferns based on the final shape that is formed when matured; these include tuberous (Ophioglossaceae, *Actinostachys*, *Lophidium*, and *Stromatopteris*), filamentous (*Schizaea* and *Trichomanes*), cordate‐thalloid (a majority of the homosporous ferns), strap‐like (some species of Lomariopsidaceae and Polypodiaceae), and ribbon‐like (some species of Pteridaceae, Polypodiaceae, and Hymenophyllaceae) forms. Most gametophytes of homosporous ferns are known as the cordate‐thalloid form, which consists of a thick, median midrib (cushion) and a one‐cell‐thick, semicircular wing. Additionally, they tend to experience rapid development by season, except in the primitive homosporous ferns (Nayar & Kaur, 1971). In contrast to the cordate‐thalloid form, the noncordate form of gametophytes has a relatively slower growth rate (Nayar & Kaur, 1971).

The fertilization of ferns, which is the mating between the spermatozoid produced by the antheridium and the egg originated from the archegonium, results in the sporophyte (Raghavan, 2005). However, certain gametophytes produce large and long‐lived populations without producing sporophytes and reproduce by asexual means, such as generating gemmae (Farrar, 1967). Rumsey and Sheffield (1996) classified these independent gametophytes into two types, obligate and facultative independent gametophytes according to the absence/presence of related sporophytes. Regarding the obligate independent gametophytes, the mature sporophyte generation is either unknown or has been found rarely as stunted juveniles. Conversely, facultative independent gametophytes occur over the geographical zone in which functional adult sporophytes are produced (Rumsey & Sheffield, 1996). Recently, Kuo et al. (2017) proposed a new definition for identifying the independence of fern gametophyte at the population level. Based on their findings, obligate independent gametophyte can flourish by asexual reproduction and do not share a geographic range with functional conspecific sporophytes. However, facultative independent gametophyte can be derived from dispersed spores of functional conspecific sporophytes and inhabits areas near the range of the functional conspecific sporophyte.

Studies on the independent gametophytes of ferns are well‐established in the North American taxa (Dassler & Farrar, 1997; Duffy, Stensvold, & Farrar, 2015; Ebihara, Farrar, & Ito, 2008; Farrar, 1967, 1974, 1978, 1985, 1992; Farrar & Mickel, 1991; Pinson, Chambers, & Sessa, 2017; Pinson & Schuettpelz, 2016; Raine, Farrar, & Sheffield, 1991). Building on these achievements, studies have been extended to the European (Li et al., 2009; Makgomol & Sheffield, 2001; Rumsey, Jermy, & Sheffield, 1998; Rumsey, Sheffield, & Farrar, 1990) and Asian (Ebihara et al., 2013, 2019; Kuo et al., 2017) taxa. These studies provide a better understanding of the distribution and habitat of independent gametophytes, thereby making it possible to tackle the problem of identifying the relationship between sporophytes and gametophytes. In the case of the Korean fern taxa, only three independent gametophytes with ribbon‐like or filamentous forms have been reported (Lee & Lee, 2015) using DNA barcoding techniques (data not shown and unpublished) without detailed information on their distribution. To our knowledge, there is no study to confirm whether the corresponding sporophytes are found close to the independent gametophytes. Therefore, it is necessary to conduct further studies to gain a better understanding of the independent gametophytes in Korea.

As the first step in understanding the independent gametophytes of the Korean fern taxa extensively, we opted to focus on the Jeju Island which was created by volcanic activity. It is located in the southwestern part of the Korean peninsula and has different elevation levels ranging from lowland to alpine (Kongk & Watts, 1993), with higher annual temperature and precipitation (Kim, Jang, Baek, Choi, & Kwon, 2006). Jeju Island was located on the land bridge between East China and Japan which were connected during the glacial periods (Harrison, Yu, Takahara, & Prentice, 2001a; Xie, 1997). As a result, the ferns distributed on the Jeju Island account for 76% of the Korean ferns and there are many families inhabiting this island alone and not found on the Korean mainland (Moon, 2008).

The island might be a suitable location for studying fern gametophytes, as the portion of epiphytic species with gemmiferous gametophytes on islands is higher than that found on the mainland, despite the near equivalency between the percentage of total epiphytes on the island and the mainland (Dassler & Farrar, 2001). In addition, all sporophytes with noncordate form gametophytes of the Korean ferns are found on Jeju Island. Therefore, it was supposed that most of the independent gametophytes, including three previously reported taxa, could be found on Jeju island.

In the present study, we aimed to identify the fern gametophytes on Jeju Island to (a) discover unknown independent gametophytes in Korea, (b) compare and identify their corresponding sporophytes and place within a phylogenetic framework, and (c) reveal crucial information regarding the distribution of gametophytes in the East Asian fern flora.

As is well known, fern gametophytes have only a few morphological characteristics and this makes distinction among congeneric gametophytes complicated. The DNA barcoding technique has opened a new era in the identification of a conspecies or closely related taxa against query species in ferns despite several controversies remaining regarding the application of this technique in angiosperms (CBOL Plant Working Group et al., 2009; Ebihara, Nitta, & Ito, 2010; de Groot et al., 2011; Li et al., 2011; Wang, Lu, Wen, Ebihara, & Li, 2016). In practice, the identification of fern gametophytes using DNA sequences has been successively reported (Chen, Huang, Kuo, Nguyen, et al., 2013; Duffy et al., 2015; Ebihara et al., 2013; Li et al., 2009; Nitta, Meyer, Taputuarai, & Davis, 2017; Schneider & Schuettpelz, 2006). In the present study, we employed two haploid markers, *rbcL* and *trnL‐F*, to identify and investigate the gametophytes occurring in Korea.

Till now, it was reported that sporophytes of *Hymenophyllum wrightii* Bosch are primarily distributed in Korea and Japan; however, a rare occurrence in the northwest coastal region of North America has been reported. It is very interesting to consider that gametophytes have been widely found throughout the northwest coastal region of North America (Duffy et al., 2015). Therefore, we briefly discuss the biogeography of *H. wrightii* to understand the evolution of this species, including the sporophytes and gametophytes.

## 2 MATERIALS AND METHODS

### 2.1 Gametophyte sampling and observation of the morphological characteristics

**FIGURE 1:**
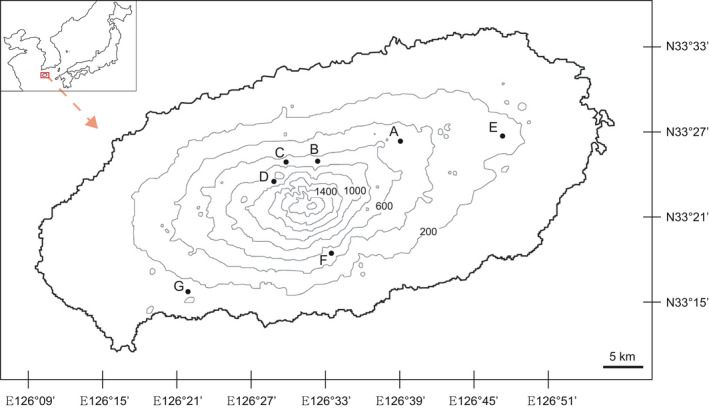
Collection sites utilized in the present study

Fourteen gametophyte populations were collected from seven sites on Jeju Island (Figure 1, Table S1). In the present study, a gametophyte population was defined as all individuals on the same rock face, except at site G in which a relatively smaller number of individuals were scattered. All living samples were transported to the laboratory at the Chungbuk National University and were grown on filter paper moistened with distilled water in a Petri dish. For magnified observation, permanent slide samples were prepared with a few individuals from each population using the Entellan^®^new (Merck). The voucher specimen was deposited in the slide box with the same collection number as that of the living materials. Their morphological characteristics were closely observed with a microscope (Olympus BX50).

### 2.2 DNA extraction and PCR amplification

As a single gametophyte was too small for the extraction of a sufficient amount of DNA for applying DNA barcoding technique, individuals within 1 × 1 cm were considered to be a clonal patch. According to this criterion, 10 prothalli near the individuals used for voucher specimens were selected from one population. Prothalli were washed with sterile distilled water and put into a 2‐ml tube with 2 mm beads. Thereafter, they were crushed in a homogenizer via three cycles for 30 s followed by 10 s at the speed 2 m/s. Genomic DNA was extracted from the homogenized sample using a DNeasy Plant Mini Kit (Qiagen), following the manufacturer's protocol.

PCR amplifications for two molecular markers were performed using Ex Taq (Takara). The reaction mixture comprised 1 U *Taq*, 2 μl of 10× buffer, 2 mM MgCl_2_, 0.8 mM dNTP, 2 μl of each primer (10 μM), 0.5 μl DNA, and distilled water filled to a total of 20 μl. Primers for the *rbcL*, *rbcLF1F,* and *rbcL1379R* (Little & Barrington, 2003) were first used for all gametophytes; however, it was not successful in the sample collected at site G. Thus, a new primer set was designed for this sample: *Pteridaceae_rbcLF* (5′‐CCACAAACGGAGACTAAAGC‐3′) and *Pteridaceae_rbcLR* (5′‐GCAGCCAATTCTGGACTCCA‐3′). For the *trnL‐F* region, primer *F* (Taberlet, Gielly, Pautou, & Bouvet, 1991) and *FernL 1Ir1* (Li, Kuo, Huang, Chiou, & Wang, 2010) were applied. The following PCR condition was employed: 95°C for 5 min, followed by 35 cycles of 95°C for 45 s, 48°C for 45 s, 72°C for 75 s, and 72°C for 5 min. PCR products were purified using a PCR purification kit (Geneall) and sequenced using the ABI 3730xl System (Macrogen). The *trnL‐F* region of *Loxogramme* and *Pleurosoriopsis* sporophytes in Korea was also amplified owing to the short length of the *Loxogramme* sporophyte sequence and the absence of the *Pleurosoriopsis* sporophyte sequence in GenBank.

### 2.3 Molecular identification of gametophytes

To identify gametophytes, we applied the sequence similarity‐based and phylogeny‐based approach using the *rbcL* and *trnL‐F* regions. Each sequence of gametophytes was compared to the nucleotide collection using BLASTn (Altschul, Gish, Miller, Myers, & Lipman, 1990) with 11 as the word size and 0.1 as the expected threshold. The following conditions were established to match the gametophytes to their counterpart sporophytes: (a) maximum percent identity, ≥99%; (b) query coverage, ≥95%; and (c) e^−5^, expected threshold (Table 1). Based on the BLASTn results, the putative sporophyte for each gametophyte was selected. Thereafter, the sequences of two markers from the subfamily or genus of the putative sporophytes were downloaded from GenBank. Best fit models of nucleotide substitution were determined using ModelFinder (Kalyaanamoorthy, Minh, Wong, von Haeseler, & Jermiin, 2017), and phylogenetic inferences by maximum likelihood were performed with IQ‐TREE (Nguyen, Schmidt, von Haeseler, & Minh, 2015) using 1,000 ultrafast bootstraps (Hoang, Chernomor, von Haeseler, Minh, & Vinh, 2017) (Figures S1–S8).

**TABLE 1:** List of sequences generated in the present study and blast search results for gametophytes using two haplotype markers

Region	Life form	Specimen	Accession	Result of BLAST			
				Matched species	Accession^a^	Percent of identity	Matched sporophyte in Jeju island
*rbcL*	Gametophyte	CBNU2018‐0317	MN868656	*Hymenophyllum wrightii*	MN583337	100.00%	Presence
		CBNU2018‐0320	MN868657	*Hymenophyllum wrightii*	MN583337	100.00%	Presence
		CBNU2018‐0322	MN868658	*Hymenophyllum wrightii*	MN583337	100.00%	Presence
		CBNU2018‐0323	MN868659	*Hymenophyllum wrightii*	MN583337	100.00%	Presence
		CBNU2018‐0324	MN868660	*Pleurosoriopsis makinoi*	KF909057.1	100.00%	Presence
		CBNU2019‐0326	MN868661	*Pleurosoriopsis makinoi*	KF909057.1	100.00%	Presence
		CBNU2018‐0331	MN868662	*Hymenophyllum wrightii*	MN583337	100.00%	Presence
		CBNU2018‐0335	MN868663	*Hymenophyllum barbatum*	MN583334	100.00%	Presence
		CBNU2018‐0337	MN868664	*Hymenophyllum barbatum*	MN583334	100.00%	Presence
		CBNU2018‐0339	MN868665	*Hymenophyllum barbatum*	MN583334	100.00%	Presence
		CBNU2018‐0357	MN868666	*Loxogramme grammitoides*	AB575275.1	100.00%	Presence
		CBNU2018‐0361	MN868667	*Loxogramme grammitoides*	MF450445.1	99.70%	Presence
		CBNU2018‐0387	MN868668	*Antrophyum obovatum*	KX164966	99.90%	Absence^b^
		CBNU2019‐0181	MN868669	*Hymenophyllum wrightii*	MN583337	100.00%	Presence
	Sporophyte	KHB1426306 (*Loxogramme grammitoides*)	MN912808				
*trnL‐F*	Gametophyte	CBNU2018‐0317	MN912790	*Hymenophyllum wrightii*	MN583337	100.00%	Presence
		CBNU2018‐0320	MN912791	*Hymenophyllum wrightii*	MN583337	100.00%	Presence
		CBNU2018‐0322	MN912792	*Hymenophyllum wrightii*	MN583337	100.00%	Presence
		CBNU2018‐0323	MN912793	*Hymenophyllum wrightii*	MN583337	100.00%	Presence
		CBNU2018‐0324	MN912794	*Pleurosoriopsis makinoi*	MN912803	99.60%	Presence
		CBNU2019‐0326	MN912795	*Pleurosoriopsis makinoi*	MN912803	99.60%	Presence
		CBNU2018‐0331	MN912796	*Hymenophyllum wrightii*	MN583337	100.00%	Presence
		CBNU2018‐0335	MN912797	*Hymenophyllum barbatum*	MN583334	100.00%	Presence
		CBNU2018‐0337	MN912798	*Hymenophyllum barbatum*	MN583334	100.00%	Presence
		CBNU2018‐0339	MN912799	*Hymenophyllum barbatum*	MN583334	99.70%	Presence
		CBNU2018‐0357	MN912800	*Loxogramme grammitoides*	MN912807	100.00%	Presence
		CBNU2018‐0361	MN912801	*Loxogramme grammitoides*	MN912807	100.00%	Presence
		CBNU2018‐0387	MN912802	*Antrophyum obovatum*	KY101389.1	99.90%	Absence^b^
		CBNU2019‐0181	MN912804	*Hymenophyllum wrightii*	MN583337	100.00%	Presence
	Sporophyte	KHB1426306 (*Loxogramme grammitoides*)	MN912807				
		KHB1038376 (*Loxogramme salicifolia*)	MN912805				
		KHB1122073 (*Loxogramme duclouxii*)	MN912806				
		CBNU2019‐0140 (*Pleurosoriopsis makinoi*)	MN912803				

^a^ Accession which was best‐matched to gametophyte sequence based on the prior condition mentioned by materials and methods.

^b^ Absence within Korea.

### 2.4 Biogeographical study of *H. wrightii*

For the biogeographical study of *H. wrightii*, the *rbcL* sequences of *H. wrightii* were retrieved from GenBank (Table 2) and used to build a haplotype network via the TCS network (Clement, Snell, Walker, Posada, & Crandall, 2002) using POPART (Leigh & Bryant, 2015).

**TABLE 2:** Sequence information used for biogeography of *Hymenophyllum wrightii*

Accession	Collection site	Generation	Reference
AB064294	Nagano, Japan	Sporophyte	Ebihara et al. (2002)
AB083277	Nagano, Japan	Sporophyte	Ebihara, Iwatsuki, Ohsawa, and Ito (2003)
MN266600	Akita prefecture, Japan	Sporophyte	Vasques, Ebihara, Hirai, Prado, and Motomi (2019)
MN266602	Tokyo, Japan	Sporophyte	Vasques et al. (2019)
MN266603	Gunma prefecture, Japan	Sporophyte	Vasques et al. (2019)
AB574724	Akita prefecture, Japan	Sporophyte	Ebihara, Iwatsuki, Ohsawa, and Ito (2010)
JN585964	Kruzof Island near Sukoi Inlet, Alaska	Gametophyte	Duffy et al. (2015)
JN585965	Big Trees Trail, Meares Island, British Columbia	Gametophyte	Duffy et al. (2015)
JN585966	Shi‐Shi Beach Trail near Neah Bay, Washington	Gametophyte	Duffy et al. (2015)
2018‐0317	Nohyeong‐dong, Jeju island	Gametophyte	In this study
2018‐0320	Haean‐dong, Jeju island	Gametophyte	
2018‐0322	Haean‐dong, Jeju island	Gametophyte	
2018‐0323	Haean‐dong, Jeju island	Gametophyte	
2018‐0331	Haean‐dong, Jeju island	Gametophyte	
2019‐0181	Tamra valley, Jeju island	Gametophyte	
2018‐0330	Haean‐dong, Jeju island	Sporophyte	MN583337 (unpublished)

## 3 RESULTS

### 3.1 Habitats of gametophytes on Jeju Island

**FIGURE 2:**
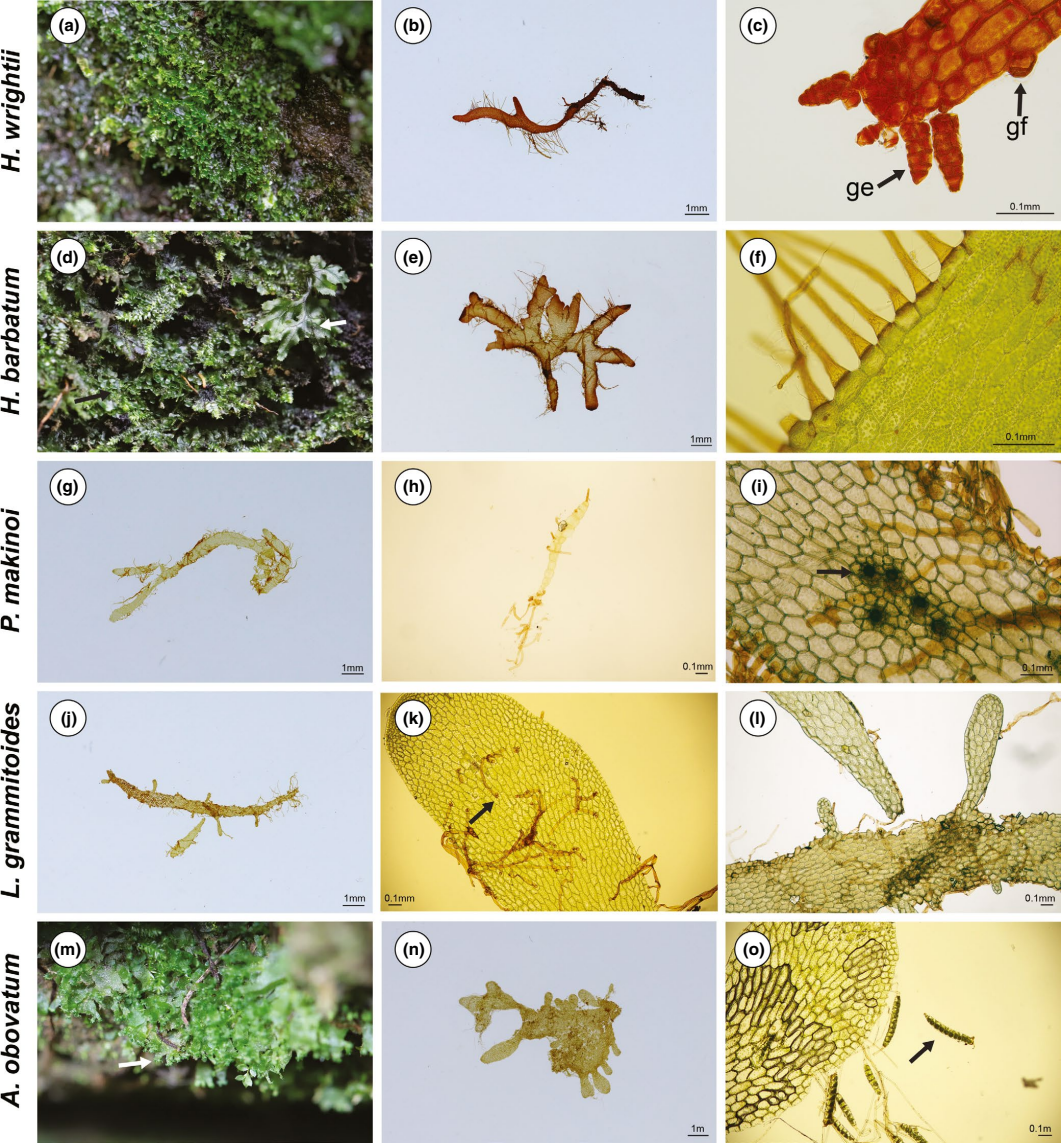
Habitat and features of long‐lived gametophytes. a–c. *Hymenophyllum wrightii*. a: Gametophytes on rocks with gemmae. b: Voucher specimen. c: Microscopic view of gemma (ge) and gemmifer (gf). d–f. *H. barbatum*. d: Gametophytes (black arrow) growing with the sporophyte (white arrow) on rocks. e: Voucher specimen. f: Growth of rhizoid on the edge of the thallus. g–i. *Pleurosoriopsis makinoi*. g: Voucher specimen. h: Gamma. i: Microscopic view of archegonia (black arrow). j–l. *Loxogramme grammitoides*. j: Voucher specimen. k: Rhizoids grew from the central area of the thallus (black arrow) and its edge. l: Irregular lobes formed from the marginal meristem. m–o. *Antrophyum obovatum*. m: Gametophytes on the steep slope with gemmae (white arrow). n: Voucher specimen. o: Microscopic view of gemma (black arrow)

Most of the gametophytes were found on rocks in wet and shaded environments. However, one population of gametophytes grew on the underside of a stump located on a trail in a valley lacking a canopy (Figure 1; site B) and another grew on a steep slope in a relatively dry valley (Figure 1; site G). Interestingly, the sporophytes of Hymenophyllaceae members frequently found nearby where gametophytes inhabited; however, gametophytes and sporophytes were physically separated except in site F in which gametophytes and sporophytes of *H. barbatum* were mixed on the same rock (Table S1; Figure 2d). In the field, half of the gametophytes contained gemmae; however, they were detached from gametophytes during transportation to the laboratory and did not recur.

### 3.2 Identification based on the molecular markers

Based on the BLAST search performed with *rbcL* and *trnL‐F*, 13 gametophytes were identified as belonging to five species known to occur in Jeju, Korea. However, the terrestrial gametophyte that occurred at the steep slope in the dry valley (site G) was matched to *Antrophyum obovatum* Baker which has not been previously recorded in Korea (Table 1). According to the phylogenetic relationship, all gametophytes formed a clade with their putative sporophytes and these nodes were supported by a strong bootstrap value (Figures S1–S8).

### 3.3 Morphological characteristics of gametophytes

All gametophytes displayed ribbon‐like forms but had slight differences according to their morphological characteristics. Gametophytes of two *Hymenophyllum* species had dark brown rhizoids at the margin and significant branching at the apex (Figure 2a–f). The only difference between the two gametophytes was the existence of gemmae. Numerous gemmae were observed at the margin of *H. wrightii* gametophytes; however, they were not found on gametophytes of *H. barbatum* (Bosch) Baker . Gametophytes of *Pleurosoriopsis makinoi* (Maxim. ex Makino) Fomin were rarely branched relative to those of *Hymenophyllum* with light brown rhizoids at the margin (Figure 2g). These gametophytes had a round apex, with some thalli containing archegonia in the center (Figure 2i); however, antheridia were not observed. Gametophytes of *Loxogramme grammitoides* (Baker) C. Chr. were branched at the margin of the thallus instead of at the apex. Marginal branches elongated and formed many lobes. After the mature lobes detached from the parental thallus, they developed into new thalli (Figure 2l). Light brown rhizoids grew from the central and marginal cells (Figure 2k). Gametophytes of *A. obovatum* had numerous gemmae that were irregularly branched (Figure 2n) and contained whitish brown rhizoids at the margin (Figure 2o).

### 3.4 Geographical variation in *H. wrightii*

**FIGURE 3:**
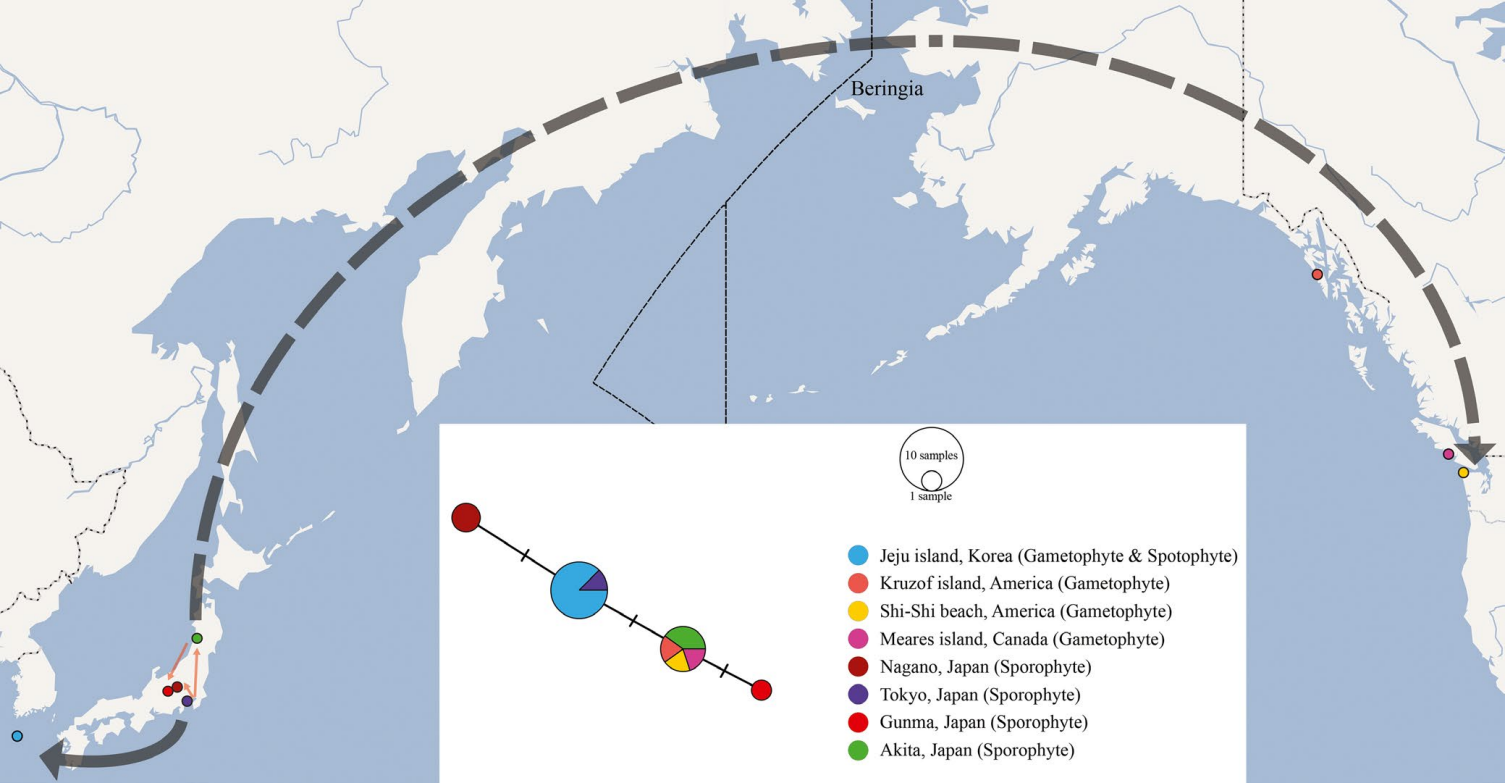
Haplotype network conducted via the TCS network (Clement et al., 2002) using POPART (Leigh & Bryant, 2015): Network view (white box) and map view (background). Thick black lines and thin red lines refer to long‐ and short‐distance dispersals, respectively

A total of four haplotypes based on the *rbcL* sequence data were found in the *H. wrightii* populations of Korea, Japan, and North America. The *rbcL* sequence of the *H. wrightii* gametophytes collected in this study was identical to that of sporophytes collected from Jeju, Korea, and Tokyo, Japan (Figure 3). Sporophytes collected from other regions in Japan, namely Nagano, Gunma, and Akita, had one or two base substitutions compared to that in sporophytes from Tokyo. Interestingly, the gametophytes occurring in North America were genetically identical to the sporophytes collected in Akita, Japan.

## 4 DISCUSSION

We found five ribbon‐like and one filamentous type gametophytes in the present study. Morphologically, the latter was similar to the filamentous gametophytes in Hymenophyllaceae (Yoroi, 1972); however, these did not form a patch of numerous gametophytes. To be verified as independent gametophytes, they should reproduce asexually for many generations and consequently form a clonal colony. In addition, *Vandenboschia* species was found nearby filamentous gametophytes. Therefore, we excluded these filamentous gametophytes from our discussion as they were supposed to be derived from spores based on the population size. In the case of the gametophytes of *H. barbatum*, the lack of a gemma was consistent with the field observation of Yoroi (1972) and Atsushi Ebihara et al. (2019). The gametophyte populations of *H. barbatum* have been maintained on Jeju Island for many years (personal communication with Mr. Lee who first found this site); however, it remained unclear whether *H. barbatum* gametophytes were independent gametophytes because we could not find any asexual reproductive organs at the research site. We also found that they intermingled with their sporophytes on the same rocks. Based on these viewpoints, we excluded this gametophyte from analysis and will conduct further studies at this site to confirm whether they are independently maintained.

Among the remaining four ribbon‐like gametophytes, *H. wrightii* and *P. makinoi* have been previously reported in Korea (Lee & Lee, 2015). One of our new findings is the first report of *L. grammitoides* in Korea as an independent gametophyte. All sporophytes corresponding to these three gametophytes occur in Jeju Island, Korea. The most interesting finding of this study is the presence of the independent gametophyte of *A. obovatum*. The independent gametophyte of this species has not been reported in nature, and even *Antrophyum* has not been found in Korea until now. Therefore, we concluded that the gametophyte of *A. obovatum* might be an “obligate independent gametophyte,” whereas the remaining gametophytes might be “facultative independent gametophytes.”

Based on our sampling experience during this study, at least on Jeju Island, it is likely that independent gametophytes have similar microhabitats as taxa belong to Hymenophyllaceae (Table S1) such as low light levels and high humidity. Further investigation will clarify environmental factors required for the establishment of independent gametophyte populations on Jeju Island.

### 4.1 Antrophyum obovatum

Although there are some reports on the independent gametophytes of vittarioids (Farrar, 1978; Farrar & Mickel, 1991; Kuo et al., 2017; Pinson & Schuettpelz, 2016; Rumsey & Sheffield, 1996), here, we present the first report of the independent gametophytes of *A. obovatum*, a member of the vittarioids. The sporophytes of *A. obovatum* are distributed in Bhutan, India, Nepal, Myanmar, Thailand, Vietnam, southern China, Taiwan, and Japan (Boufford et al., 2003; Zhang, 1998), but not in Korea. It is likely that the low sea level during the glacial periods was linked to the occurrence of the present flora of East China and Japan (Harrison, Yu, Takahara, & Prentice, 2001b; Ijiri et al., 2005; Qian & Ricklefs, 2000; Xie, 1997) and numerous ferns migrated from China and Taiwan to Japan (Guo, Kato, & Ricklefs, 2003; Iwatsuki, 1994). It is also possible that ferns were independently moved from China to Japan after glaciation by long‐distance dispersal. However, in terms of parsimony, the former is a more simple and adequate to interpretation of why most vittarioid ferns occurring in Japan are also found in China and Taiwan (Chen, Ebihara, Chiou, & Li, 2014; Chen, Huang, Kuo, Chang, et al., 2013; Ebihara, 2016; Wu, Raven, & Hong, 2013) (Table 3). While their migration from China to Japan during the glacial periods (Harrison et al., 2001b; Ijiri et al., 2005; Qian & Ricklefs, 2000), many sporophytes and gametophytes of ferns might have sought suitable habitats to settle throughout the land bridge (in which Jeju island is included) between China and Japan. From this perspective on the route of migration, it is interesting that only the sporophyte of *Haplopteris flexuosa* (Fée) E.H. Crane was reported on Jeju Island from among the vittarioids that appear in Japan. This means that Jeju Island might therefore be unsuitable for vittarioid sporophytes, which generally comprise tropical or subtropical ferns (Kramer, 1990).

**TABLE 3:** List of vittarioid ferns distributed in East Asia

Subfamily	Species	Korea	Japan	China	Taiwan
Vittarioideae	*Antrophyum annamense*	x	x	o	x
	*Antrophyum callifolium*	x	x	o	x
	*Antrophyum castaneum*	x	x	x	o
	*Antrophyum formosanum*	x	o	x	o
	*Antrophyum henryi*	x	x	o	o
	*Antrophyum obovatum*	o^a^, ^b^	o	o	o
	*Antrophyum parvulum*	x	x	o	o
	*Antrophyum sessilifolium*	x	x	x	o
	*Antrophyum vittarioides*	x	x	o	x
	*Antrophyum wallichianum*	x	x	o	x
	*Haplopteris amboinensis*	x	x	o	x
	*Haplopteris anguste‐elongata*	x	o	o	o
	*Haplopteris doniana*	x	x	o	x
	*Haplopteris elongata*	x	x	o	o
	*Haplopteris flexuosa*	o^a^	o	o	o
	*Haplopteris fudzinoi*	x	o	o	x
	*Haplopteris hainanensis*	x	x	o	x
	*Haplopteris heterophylla*	x	x	o	o
	*Haplopteris himalayensis*	x	x	o	x
	*Haplopteris linearifolia*	x	x	o	x
	*Haplopteris mediosora*	x	o	o	o
	*Haplopteris plurisulcata*	x	x	o	o
	*Haplopteris sikkimensis*	x	x	o	x
	*Haplopteris taeniophylla*	x	x	x	o
	*Haplopteris yakushimensis*	x	o	x	x
	*Haplopteris zosterifolia*	x	o	o	o
	*Vaginularia paradoxa*	x	x	x	o
	*Vaginularia trichoidea*	x	x	o	o

^a^ Restrict to Jeju island in Korea.

^b^ Gametophyte only.

Because of the cold, winter monsoon and the Halla mountain at the center of Jeju island, the northern part of Jeju is colder than the southern region. Especially, Halla mountain is often covered with snow in the winter season. In contrast, the location of site G, where gametophytes of *A. obovatum* were found in the present study, is close to the south coast of Jeju island, which is close to the warm Tsushima Current (Dolezal et al., 2012). Owing to this geographical condition, this site is satisfactory for the inhabitation of subtropical ferns. However, it is likely that the relatively dry conditions, compared to the general habitat of vittarioids, make it unsuitable for the survival of the sporophyte of *A. obovatum*. Although we did not find experimental evidence to prove the tolerance of *A. obovatum* gametophytes to the site, it was assumed that they may better tolerate cold (Sato & Sakai, 1981) and desiccation (Watkins, Mack, Sinclair, & Mulkey, 2007) conditions than the sporophytes.

### 4.2 Hymenophyllum wrightii

According to phytogeographical affinity, Xie (1997) suggested that in ancient times, East China and South Japan were closer in proximity than Korea and Japan. This is caused by the geographical connection of Japan to East China and not to Korea during the glacial periods. However, Jeju Island was located on a land bridge from the Asian continent to Japan during this period (Harrison et al., 2001b; Ijiri et al., 2005; Qian & Ricklefs, 2000). The Halla Volcano, which reaches an altitude of almost 2,000 m a.s.l., and with proximity to the warm Tsushima Current (Dolezal et al., 2012), provides the appropriate condition for various ferns to exist on Jeju Island. In practice, all fern species, distributed on Jeju Island except for *Mankyua chejuense* (an endemic species on Jeju island), are conspecific to those in Japan and not to those on the Korean mainland (Ebihara, 2016; Moon, 2008). Therefore, it is predictable that the migration of fern spores between Jeju Island and Japan may have been more common and widespread. For example, high phytogeographical affinity between the fern flora of Jeju Island and Japan was found in *Selliguea hastata*. Allozyme data showed that no variation was found among the populations of the Korean mainland; however, polymorphisms were detected between the populations of Jeju Island and Japan (Chung et al., 2013).

In addition, closely related species of monilophytes, conspecies, or vicarious species, between North America and Japan, have been reported (Iwatsuki, 1994; Li, 1952). To explain this long‐distance distribution, it is suggested that wind might have served as the means of transportation of fern spores between the two continents via the Bering Strait or the Aleutian chain during the glacial period (Kim & Kim, 2014; Lu et al., 2011) including that of *H. wrightii* (Iwatsuki, 1994).

The sporophyte of *H. wrightii* is primarily distributed in Korea and Japan; however, it is also reported in the northwest coast of North America (Duffy et al., 2015; Iwatsuki, 1995). Compared to that for sporophytes, a relatively wider range has been reported for the gametophytes of *H. wrightii* (Duffy et al., 2015). The main difference between independent gametophytes of *H. wrightii* found in North America and those on Jeju Island is the presence of sporophytes near the location of independent gametophytes. On Jeju Island, sporophytes of *H. wrightii* were found in sites C and D and were previously reported in site B. However, Duffy et al. (2015) observed the independent gametophytes of *H. wrightii* in several different regions of North America; however, sporophytes were only observed in Haida Gwaii. Therefore, they questioned how the North American gametophyte populations could have been established. Chlorophyllous spores, such as those of *H. wrightii*, might be more susceptible to climate extremes and are shorter‐lived than achlorophyllous spores (Lloyd & Klekowski, 1970); this is despite the range of ferns in the Andean region being unaffected by the presence or absence of chlorophyll in spores (Kessler, 2002). A long dispersal range of gemmae is also unlikely as they are larger and heavier than spores.

To clarify the presence of the independent gametophytes of *H. wrightii* in different regions of North America, excluding Haida Gwaii, without sporophytes, it is necessary to explore the relationship among the populations of sporophytes and gametophytes in distinct regions. Based on haplotype analysis, the Japanese populations are genetically diverse and may have multiple refugia. As the Korean populations seem to be recolonized from one refugium in central Japan via Jeju Island (Figure 3), no genetic variation was observed in *H. wrightii* in Korea regardless of the generation (i.e., gametophyte or sporophyte). On the other hand, the North American populations were observed to be genetically identical to the north Japanese population in the Akita province.

*H. wrightii* prefers a high altitude in Korea but sea level in Northwest America. We hypothesized that *H. wrightii* was more prevalent in the low land, especially along the coast, throughout northeast Asia and North America owing to migrating from the north Japanese refugium via the Bering land bridge during the glacial periods (K Iwatsuki, 1994). However, the environmental changes, such as aridity, temperature, or light conditions (Atsushi Ebihara et al., 2019; Makgomol & Sheffield, 2001; Sato & Sakai, 1981), might quickly suppress the development of the *H. wrightii* sporophyte throughout North America, except in Haida Gwaii, an archipelago on the northern Pacific coast of Canada. Consequently, the short period that caused the sporophyte's extinction may have restricted the nucleotide substitutions between *H. wrightii* haplotypes in North America and those in south Japan. Similarly, a fast disappearance of the *H. wrightii* sporophytes was observed in mainland Korea like in Namdeugyusan, where a mountain that ranges over 80 km inland is present. Although the previously collected herbarium specimen record stated that the *H. wrightii* sporophytes were present 6 years ago, no sporophytes were found at the same site in 2019. However, colonies of gametophytes were observed under many rocks in the same area. Thus, the sporophytes at this site might either be extinct or rapidly declining, whereas the gametophytes have been retained.

### 4.3 Pleurosoriopsis makinoi

In Korea, the independent gametophyte of *P. makinoi* was first reported by Lee et al. (2014). Sporophytes of this species occur throughout the Korean mainland and Jeju island; however, their occurrence is rather sparse (Lee & Lee, 2015; Moon, 2008). Ebihara et al. (2019) observed the consequence of different tree cover over sporophytes and gametophytes. As a result, they suggested that the light condition was an important factor in preventing sporophyte formation. However, we found a patch of sporophytes within 500 m of the gametophytes. Such findings imply that in addition to the light condition, differences in microenvironments suppress sporophyte production. Further long‐term monitoring about the habitat conditions of *P. makinoi* gametophytes will assist in the better understanding for the suppression factor of *P. makinoi* sporophytes.

### 4.4 Loxogramme grammitoides

Previously, gametophytes of *L. grammitoides* were reported in Japan; however, a detailed morphological description was not provided (Ebihara et al., 2013). Nayar (1968) reported morphological differences in the young stages between the species of *Loxogramme*; the young prothalli of *L. lanceolate* become strap‐shaped, whereas those of *L. involuta* expanded irregularly to exhibit unequal marginal growth. Ultimately, these prothalli become ribbon‐like and highly branched. Based on our findings, the independent gametophytes of *L. grammitoides* were elongated but possessed fewer branches than those of *Hymenophyllum*. In contrast to *Hymenophyllum* gametophytes, marginal growth of *L. grammitoides* gametophytes resulted in many strap‐shaped lobes, which detached from the parental prothallus for development into new prothalli (Figure 2l).

As in the case of *P. makinoi*, the sporophytes of *L. grammitoides* have rapidly declined on Jeju Island (Kim et al., 2008; Moon, 2008), therefore, the record of gametophytes in this species is very important. We also failed to find sporophytes of *L. grammitoides* at previously collected sites of Jeju Island according to voucher specimen information. If extirpation of these sporophytes is complete on Jeju island, the gametophytes will be considered obligate independent gametophytes, and not facultative independent gametophytes as defined in this study.

Herein, we could not clarify whether the rapid decline in the *L. grammitoides* sporophyte population is a temporary issue caused by artificial factors, such as logging or land development, or a consequence of climate change. Nevertheless, if the latter is true, the *L. grammitoides* population on Jeju Island is in the process of changing from sporophytes to gametophytes, similar to the *H. wrightii* in North America. Additionally, its assignment to facultative independent gametophytes of *L. grammitoides* will change to that of obligate independent gametophytes according to the definition provided by Kuo et al. (2017). If the environment is perfectly suitable to the sporophytes of ferns, maintaining facultative independent gametophytes will not be necessary to sustain the population and extreme climate changes will result in the transition of facultative independent gametophytes to obligate independent gametophytes. It could be suggested that the maintenance of obligate independent gametophytes appears to be similar to that of hibernation in animals, who are awaiting the occurrence of an appropriate environment. Therefore, a comparative study of the habitats of both generations of *L. grammitoides* should be conducted to shed light on their tolerance levels. Moreover, it would help in the better understanding of how this species is suited to its current environment.

## CONFLICT OF INTEREST

The authors declare that the research was conducted in the absence of any commercial or financial relationships that could be construed as a potential conflict of interest.

## AUTHOR CONTRIBUTION

**Sang Hee Park:** Data curation (equal); investigation (equal); resources (lead); writing – original draft (equal). **Jung Sung Kim:** Funding acquisition (equal); investigation (equal); methodology (equal); supervision (equal); writing – original draft (equal); writing – review and editing (equal). **Hyoung Tae Kim:** Conceptualization (equal); formal analysis (equal); funding acquisition (equal); investigation (equal); methodology (equal); resources (equal); software (equal); supervision (equal); writing – review and editing (equal).

## Supporting information

Appendix S1

## Data Availability

All DNA sequences (Genebank accessions MN868656–MN868669 and MN912790–MN912808) generated in the present study are available in the GenBank repository.
