# Supplementary material for: Study of the independent gametophytes found on Jeju Island in South Korea and the first record of the obligate independent gametophyte of Antrophyum obovatum Baker
Source: Ecol Evol. 2020 Jul 7;10(14):7826–38. doi: 10.1002/ece3.6510 (PMC7391314; doi:10.1002/ece3.6510)
Supplement: Supplementary file 1 — Appendix S1 [file ECE3-10-7826-s001.docx]

**Appendix S1**

Phylogenetic trees used in this study were constructed by IQ-TREE (Nguyen, Schmidt, von Haeseler, & Minh, 2015). Branch lengths on the phylogenies were ignored to make presentation easier.


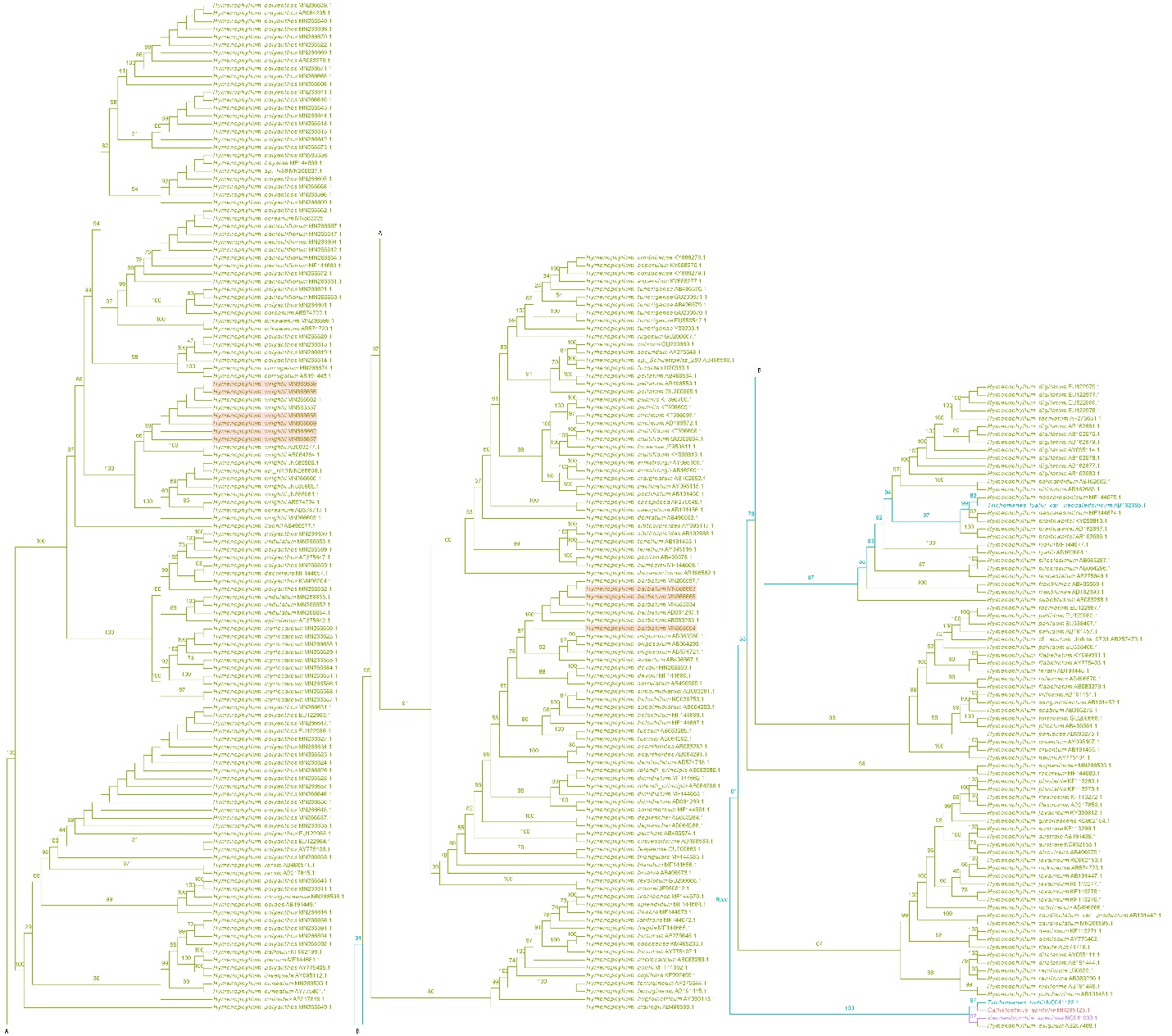


Figure S1. Phylogeny of the genus *Hymenophyllum* using Maximum likelihood with *rbcL* gene. *Trichomanes*, *Callistopteris*, and *Vandenboschia* species were used as outgroups. Numbers on the branches refer to bootstrap support of ML. Different colors of branches and external nodes refer to different genera. Gametophyte sequences are highlighted by red boxes.


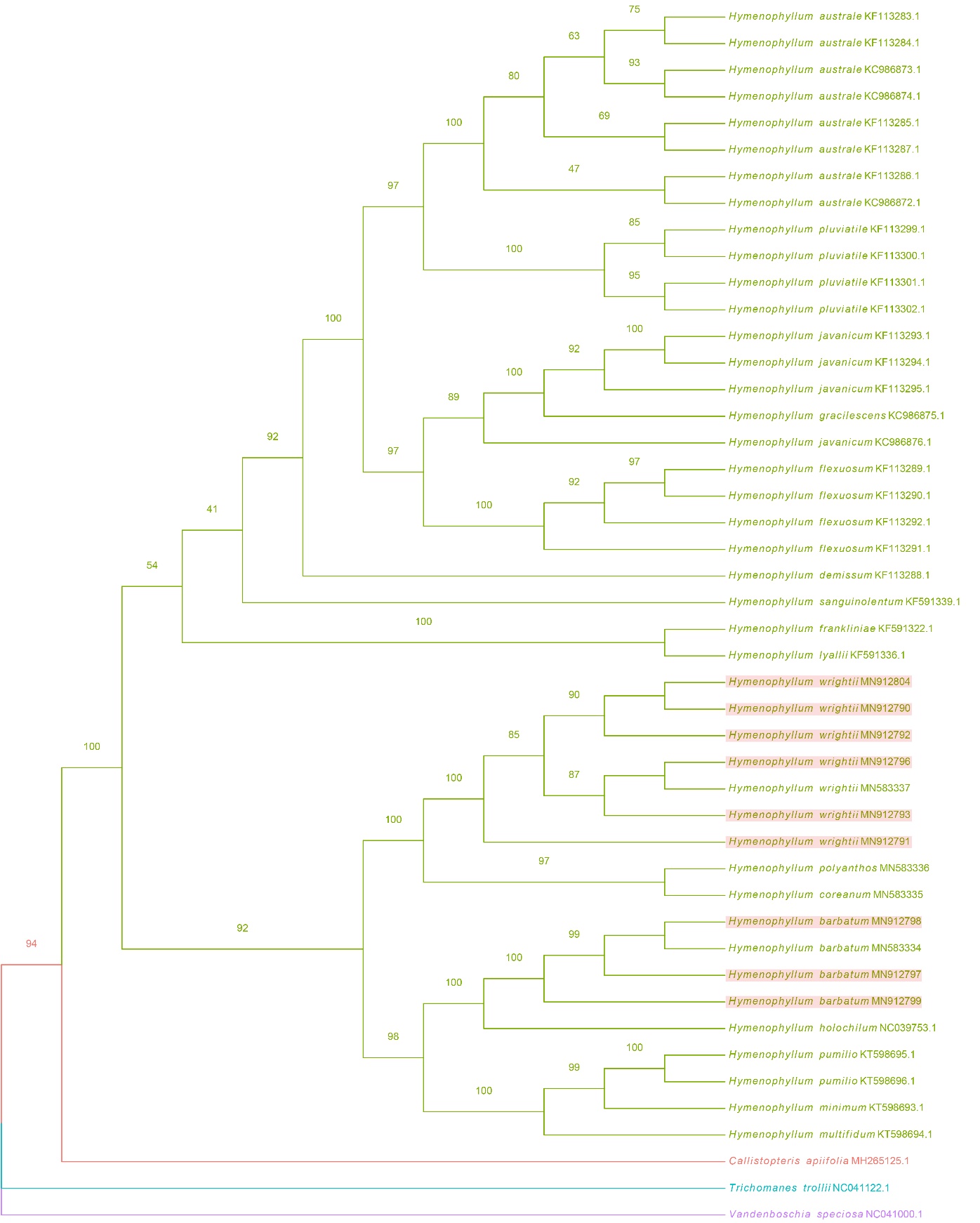


Figure S2. Phylogeny of the genus *Hymenophyllum* using Maximum likelihood with *trnL-F* IGS. *Trichomanes*, *Callistopteris*, and *Vandenboschia* species were used as outgroups. Numbers on the branches refer to bootstrap support of ML. Different colors of branches and external nodes refer to different genera. Gametophyte sequences are highlighted by red boxes.


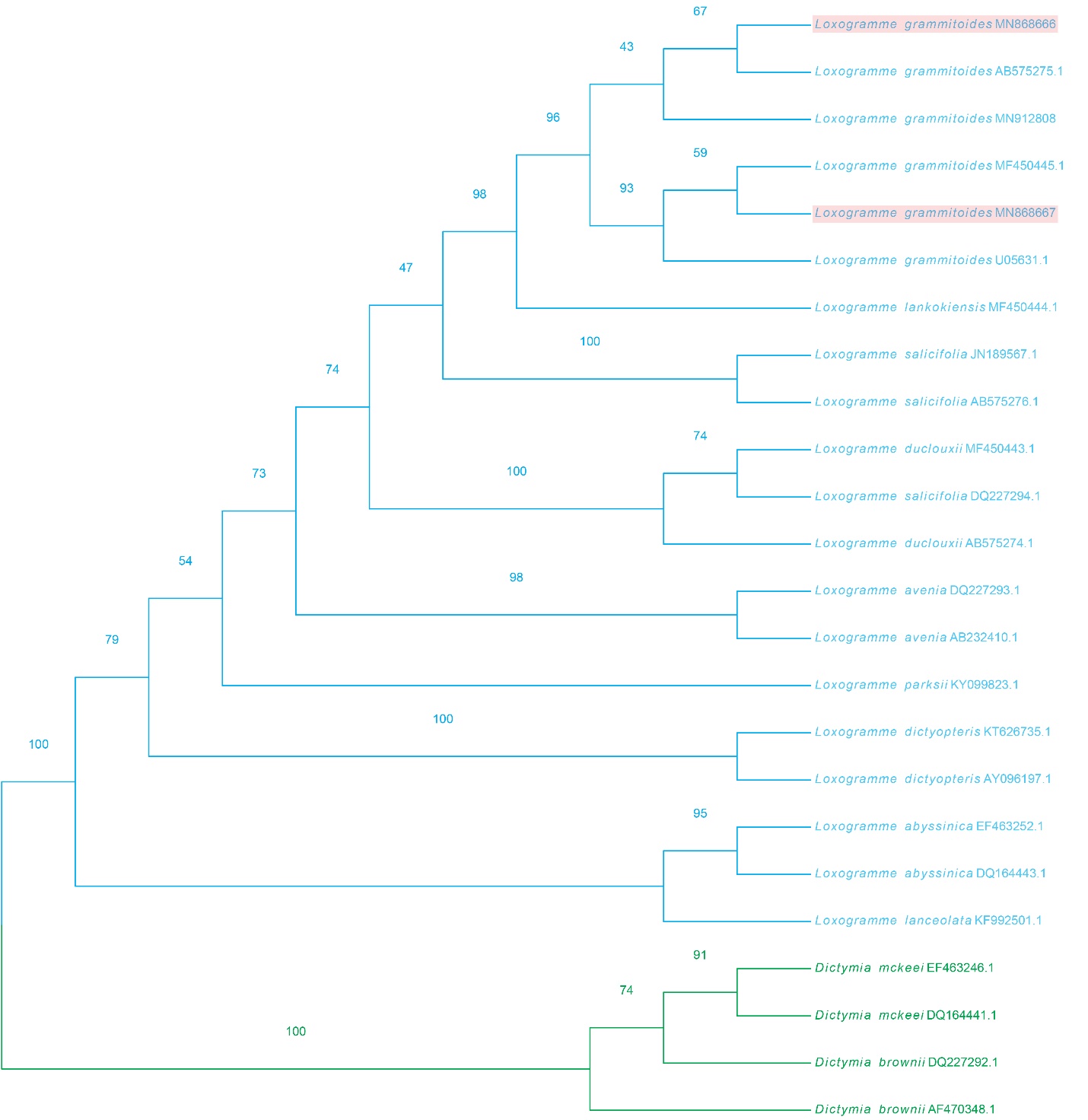


Figure S3. Phylogeny of the genus *Loxogramme* using Maximum likelihood with *rbcL* gene. *Dictymia* species were used as outgroups. Numbers on the branches refer to bootstrap support of ML. Different colors of branches and external nodes refer to different genera. Gametophyte sequences are highlighted by red boxes.


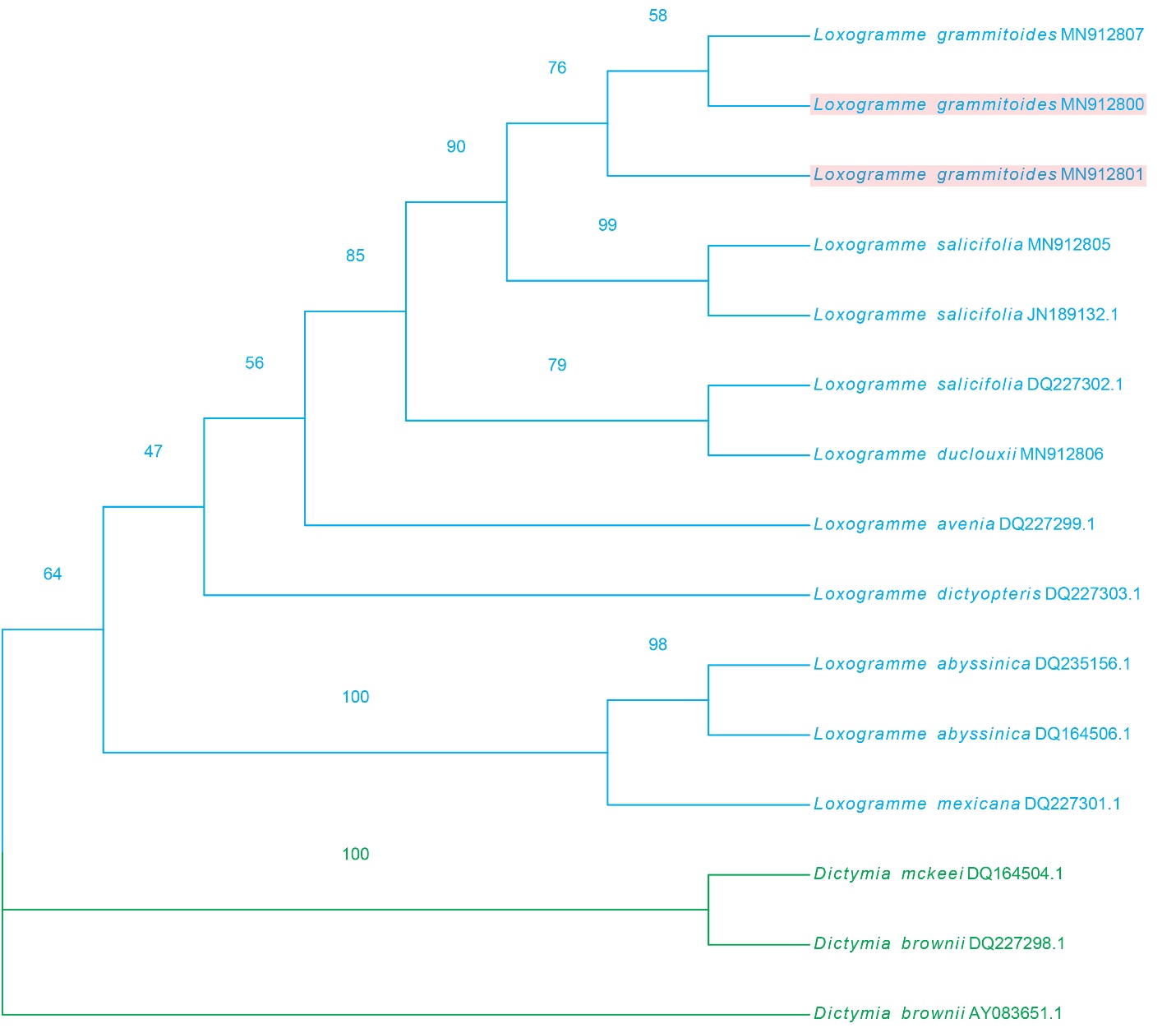


Figure S4. Phylogeny of the genus *Loxogramme* using Maximum likelihood with *trnL-F* IGS. *Dictymia* species were used as outgroups. Numbers on the branches refer to bootstrap support of ML. Different colors of branches and external nodes refer to different genera. Gametophyte sequences are highlighted by red boxes.


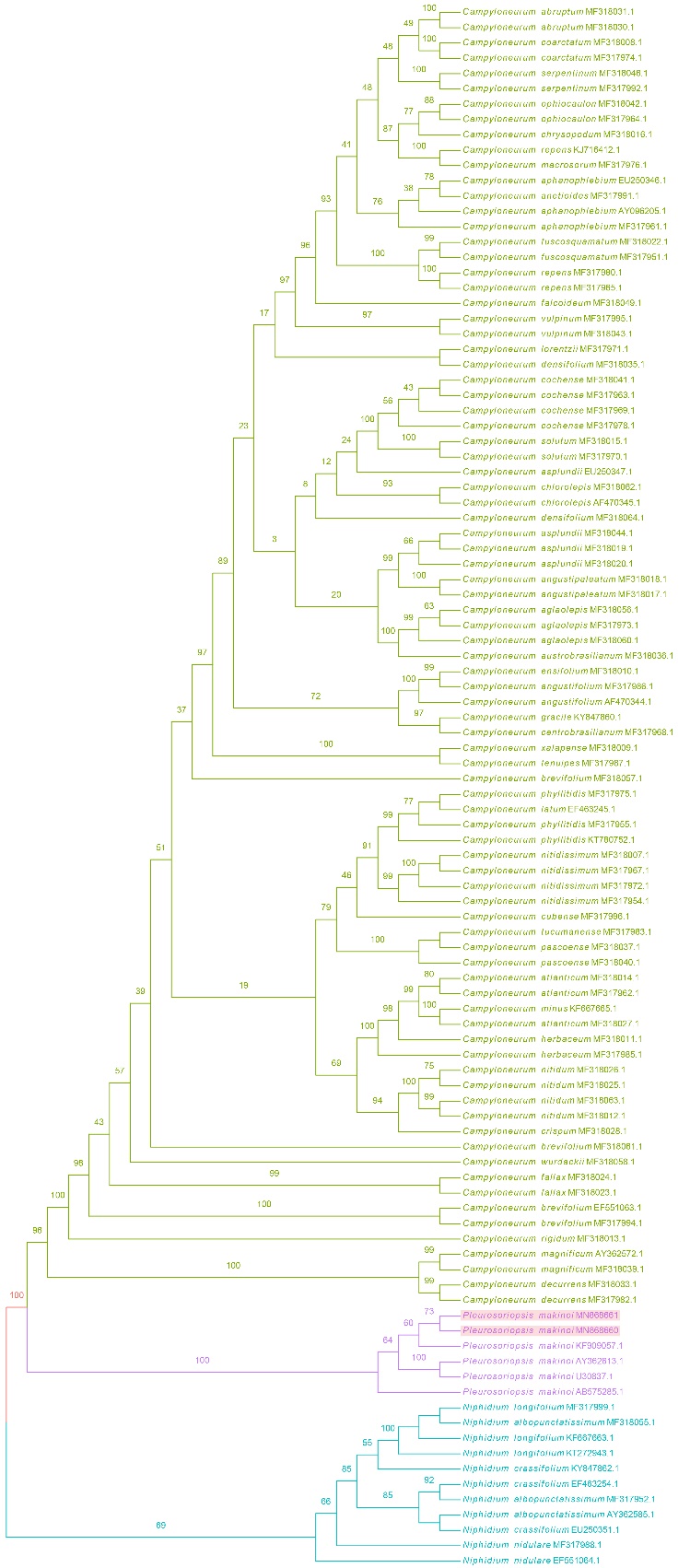


Figure S5. Phylogeny of the genus *Pleurosoriopsis* + *Campyloneurum* using Maximum likelihood with *rbcL* gene. *Niphidium* species were used as outgroups. Numbers on the branches refer to bootstrap support of ML. Different colors of branches and external nodes refer to different genera. Gametophyte sequences are highlighted by red boxes.


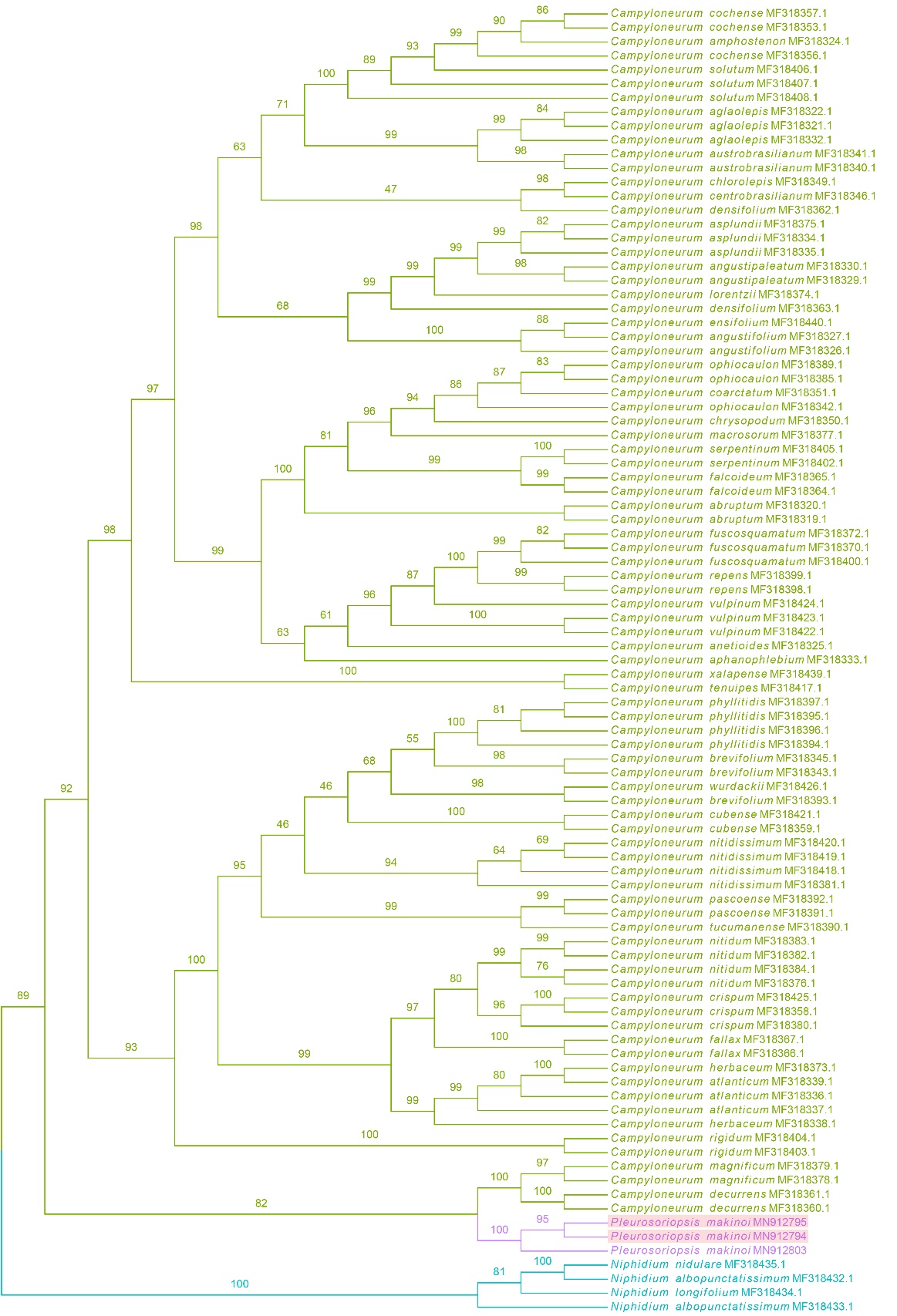


Figure S6. Phylogeny of the genus *Pleurosoriopsis* + *Campyloneurum* using Maximum likelihood with *trnL-F* IGS. *Niphidium* species were used as outgroups. Numbers on the branches refer to bootstrap support of ML. Different colors of branches and external nodes refer to different genera. Gametophyte sequences are highlighted by red boxes.


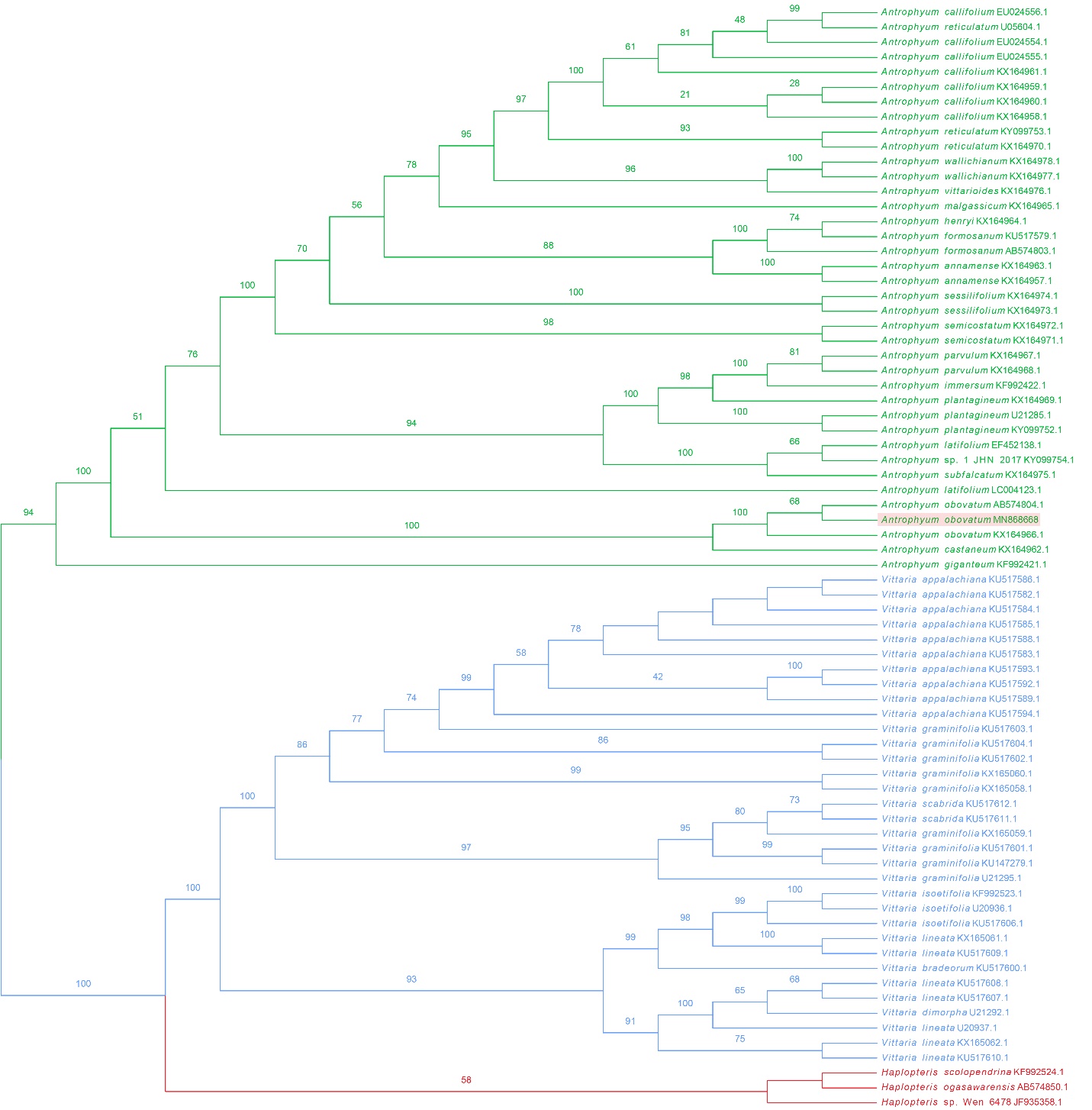


Figure S7. Phylogeny of the genus *Antrophyum* using Maximum likelihood with *rbcL* gene. *Vittaria* and *Haplopteris* species were used as outgroups. Numbers on the branches refer to bootstrap support of ML. Different colors of branches and external nodes refer to different genera. Gametophyte sequences are highlighted by red boxes.


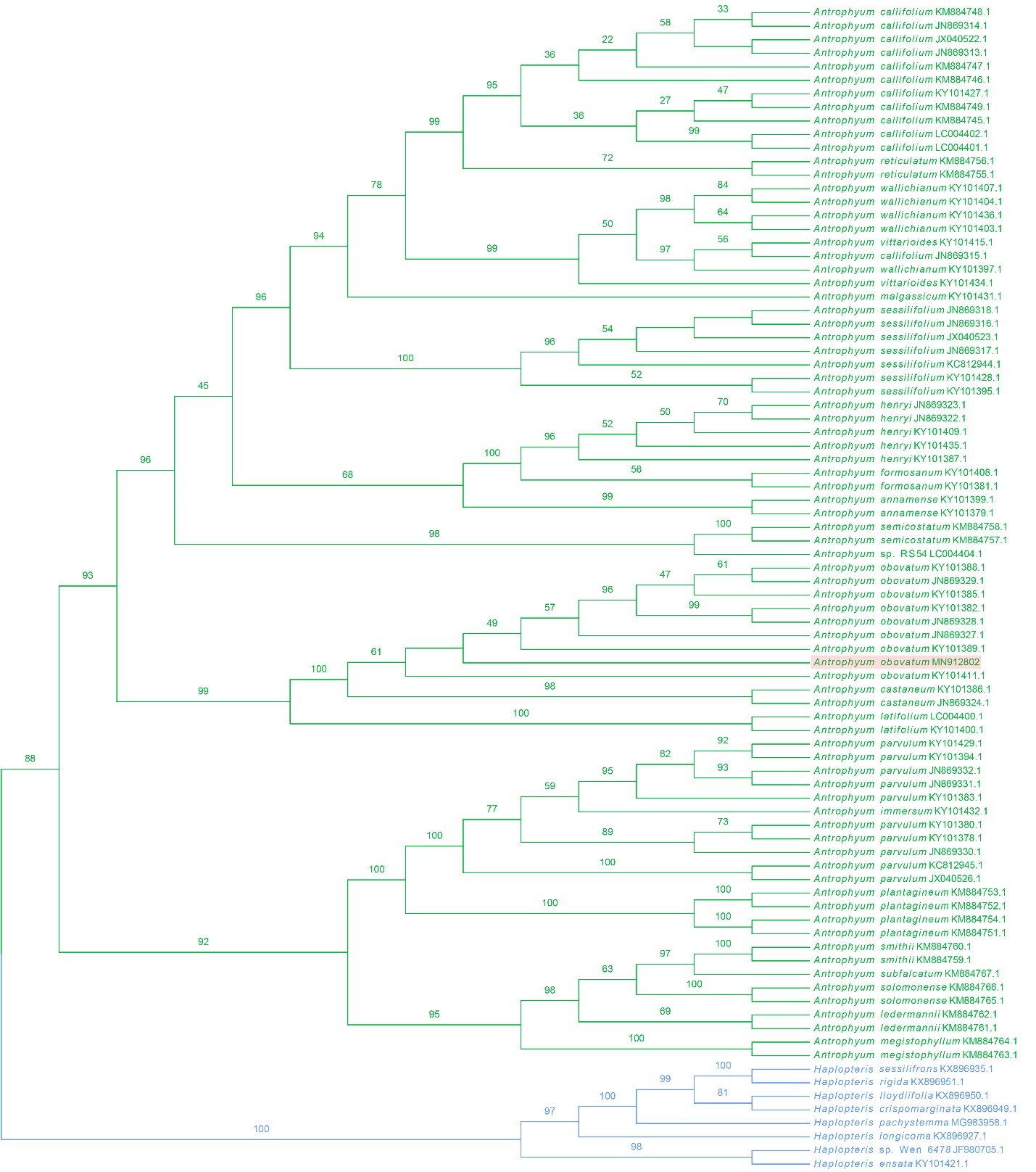


Figure S8. Phylogeny of the genus *Antrophyum* using Maximum likelihood with *trnL-F* IGS. *Haplopteris* species were used as outgroups. Numbers on the branches refer to bootstrap support of ML.

Table S1. Information on collection sites and population numbers analyzed in the present study.

| Site | No. of populations | Altitude (m) | Environment of site |
| --- | --- | --- | --- |
| A | 1 | 460 | Wet and shady depression |
| B | 1 | 706 | Wet and shady valley |
| C | 1 | 717 | Wet and shady depression |
| D | 6 | 900 | Wet and shady depression |
| E | 1 | 230 | Wet and shady depression |
| F | 3 | 496 | Wet and shady valley |
| G | 1 | 128 | Relatively dry valley |

Table S2. Features of gametophytes populations collected from 7 sampling sites.

| Substrate | Site | Voucher | Sporophyte within 1 m or less | Color of rhizoid | Gemma |
| --- | --- | --- | --- | --- | --- |
| Rock | A | CBNU2018-0361 | *Vandenboschia* ⨯ *stenosiphon* | Brown | Not found |
|  | B | CBNU2019-0181 | NA ^a^ | Brown | Observed |
|  | C | CBNU2018-0317 | *Hymenophyllum wrightii* | Brown | Observed |
|  | D | CBNU2018-0320 | *Hymenophyllum wrightii* | Brown | Observed |
|  |  | CBNU2018-0322 | *Hymenophyllum polyanthos* | Brown | Observed |
|  |  | CBNU2018-0323 | *Hymenophyllum wrightii* | Brown | Observed |
|  |  | CBNU2018-0324 | NA ^b^ | Brown | Observed |
|  |  | CBNU2018-0326 | *Crepidomanes minutum* | Brown | Not at all |
|  |  | CBNU2018-0331 | *Hymenophyllum wrightii* | Brown | Not at all |
|  | E | CBNU2018-0357 | *Crepidomanes latealatum* | Brown | Not at all |
|  | F | CBNU2018-0335 | *Hymenophyllum barbatum* | Brown | Not at all |
|  |  | CBNU2018-0337 | *Hymenophyllum barbatum* | Brown | Not at all |
|  |  | CBNU2018-0339 | *Hymenophyllum barbatum* | Brown | Not at all |
| Soil | G | CBNU2018-0387 | *Vandenboschia* ⨯ *stenosiphon* | Whitish brown | Observed |

^a^ absence of Hymenophyllaceae species within 100 m or less

^b^ presence of Hymenophyllaceae species within 5 m or less from out of the rock containing gametophytes
